# Effect of Cognitive Demand on Functional Visual Field Performance in Senior Drivers with Glaucoma

**DOI:** 10.3389/fnagi.2017.00286

**Published:** 2017-08-30

**Authors:** Viswa Gangeddula, Maud Ranchet, Abiodun E. Akinwuntan, Kathryn Bollinger, Hannes Devos

**Affiliations:** ^1^Department of Physical Therapy and Rehabilitation Science, University of Kansas Medical Center, Kansas City KS, United States; ^2^Laboratoire Ergonomie et Sciences Cognitives pour les Transports (LESCOT), IFSTTAR, TS2, Université de Lyon Lyon, France; ^3^Department of Ophthalmology, Medical College of Georgia, Augusta University, Augusta GA, United States

**Keywords:** glaucoma, cognition, psychomotor, elderly, driving

## Abstract

**Purpose:** To investigate the effect of cognitive demand on functional visual field performance in drivers with glaucoma.

**Method:** This study included 20 drivers with open-angle glaucoma and 13 age- and sex-matched controls. Visual field performance was evaluated under different degrees of cognitive demand: a static visual field condition (C1), dynamic visual field condition (C2), and dynamic visual field condition with active driving (C3) using an interactive, desktop driving simulator. The number of correct responses (accuracy) and response times on the visual field task were compared between groups and between conditions using Kruskal–Wallis tests. General linear models were employed to compare cognitive workload, recorded in real-time through pupillometry, between groups and conditions.

**Results:** Adding cognitive demand (C2 and C3) to the static visual field test (C1) adversely affected accuracy and response times, in both groups (*p* < 0.05). However, drivers with glaucoma performed worse than did control drivers when the static condition changed to a dynamic condition [C2 vs. C1 accuracy; glaucoma: median difference (Q1–Q3) 3 (2–6.50) vs. controls: 2 (0.50–2.50); *p* = 0.05] and to a dynamic condition with active driving [C3 vs. C1 accuracy; glaucoma: 2 (2–6) vs. controls: 1 (0.50–2); *p* = 0.02]. Overall, drivers with glaucoma exhibited greater cognitive workload than controls (*p* = 0.02).

**Conclusion:** Cognitive demand disproportionately affects functional visual field performance in drivers with glaucoma. Our results may inform the development of a performance-based visual field test for drivers with glaucoma.

## Introduction

Glaucoma is a progressive optic neuropathy characterized by slow degeneration of retinal ganglion cells and their axons resulting in irreversible loss of peripheral field of vision (Weinreb and Khaw, 2004; Weinreb et al., 2014). More than 70 million people worldwide are estimated to be affected by glaucoma with approximately 10% being bilaterally blind (Quigley, 2006). The usual process of the disease diagnosis includes assessment of damage to the optic disk and retinal nerve fiber layer, and clinical evaluation of the physiological visual field (Quigley et al., 1981; Weinreb and Khaw, 2004; Harwerth et al., 2010).

The Humphrey visual field analyzer (HVF) is the most commonly used method of detecting physiological visual field in individuals with glaucoma (Beck et al., 1985; Mills et al., 1986; Ballon et al., 1992; Agarwal et al., 2000; Talbot et al., 2013). The physiological visual field assessed using the HVF comprises the detection of static stimuli presented one at a time in the periphery. However, there is increasing evidence that physiological visual field defects do not reflect performance in daily-life activities such as driving (Henderson and Burg, 1974; Hills and Burg, 1977; Shinar, 1977; Wood and Troutbeck, 1995). Driving requires an individual to respond appropriately to many static and dynamic visual stimuli in cluttered environments. Outcomes on the HFV test are only moderately predictive of driving safety outcomes in glaucoma (Ball et al., 1993; Tatham et al., 2015), which may be attributed to the fact that safe driving not only requires intact physiological visual field, but also depends on the attentional capacity of the driver and the cognitive demand of the task (Owsley et al., 1998; Rubin et al., 2007; Owsley and McGwin, 2010). These three factors define the individual’s functional visual field.

Since the HVF test lacks the face validity to determine functional visual field performance while driving, the Useful Field of View (UFOV^®^) test was developed to evaluate the impact of cognitive demand on functional visual field performance (Ball et al., 1990). The size of the functional visual field is narrowed when a subject attempts to accurately detect a centrally presented stimulus while paying attention to another stimulus that is simultaneously presented in the periphery without (divided attention) and with (selective attention) additional distracters (Plude and Hoyer, 1985; Parasuraman and Nestor, 1991). The functional field of view of the UFOV^®^ test is determined by the performance in speed of processing, divided attention, and selective attention. The UFOV^®^ test shows to be more sensitive in predicting motor vehicle crashes of older drivers and also of drivers with glaucoma compared with the HFV test (Ball et al., 1993; Tatham et al., 2015). Yet, the UFOV^®^ test only evaluates 30° of horizontal field of view and does not account for the visual flow and the psychomotor activity of steering and pedal operation that is typical of driving.

The shrinkage of the functional visual field is postulated to result from an increase in cognitive demand of the task. This increased cognitive demand imposes a greater strain on the available cognitive resources, resulting in a greater cognitive workload exhibited by the subject to continue performing the task (Kahneman, 1973). Psychophysiological studies have identified several neurophysiological measures that can accurately assess the amount of cognitive workload needed to execute a task in real-time (Ranchet et al., 2017a). Task-evoked pupillary response (TEPR) accurately reflects cognitive workload through inhibition of the parasympathetic nucleus of Edinger Westphal, resulting in pupil dilation (Beatty, 1982; Eckstein et al., 2017).

Recently, studies have investigated the use of TEPR as a measure of cognitive status in individuals at risk of cognitive impairment (Wang et al., 2016; Orlosky et al., 2017). Ranchet et al. (2017b) demonstrated that cognitive workload extracted from TEPR was greater in individuals with Parkinson’s disease at risk for cognitive impairment when compared to age-matched control participants in a simple speed of processing task. The loss of peripheral field of view in glaucoma may impose a greater cognitive workload, especially under heavy cognitive demand. This increased cognitive workload may reflect a compensatory mechanism for the loss of peripheral visual field. Although the link between glaucoma and cognitive impairment remains elusive, some studies have shown a significant association between scores on a general screen of cognitive functions and visual field loss in glaucoma (Wostyn et al., 2009; Harrabi et al., 2015; Bulut et al., 2016; Diniz-Filho et al., 2017). In addition, neurodegenerative lesions have been detected in the intracranial optic nerve, lateral geniculate nucleus, and visual cortex, suggesting that glaucoma could be grouped with Alzheimer’s and Parkinson’s diseases as a neurodegenerative condition (Gupta et al., 2006, 2007). In support of this hypothesis, individuals with glaucoma are expected to exhibit greater cognitive workload compared to controls, especially under strenuous cognitive demand.

Therefore, the aim of this study was to investigate the effect of an increase in cognitive demand on the functional visual field of individuals with glaucoma while driving in a dynamic and cluttered environment. We hypothesized that (1) an increase in cognitive demand while driving in a dynamic and cluttered environment significantly alters the functional visual field of individuals with glaucoma and healthy controls, (2) functional visual field is affected more in individuals with glaucoma than in healthy controls, and (3) cognitive demand disproportionately worsens the functional visual field in individuals with glaucoma than in healthy controls.

## Materials and Methods

### Participants and Recruitment

Twenty participants with open-angle glaucoma were recruited from the Department of Ophthalmology at Augusta University, Augusta, GA, United States. Eligibility criteria included: (1) diagnosis of open-angle glaucoma exemplified by optic nerve damage and visual field loss; (2) a valid driver’s license; (3) drove at least 500 miles 1 year prior to testing; and (4) devoid of other visual, neurological, internal or psychiatric conditions that might interfere with driving. Thirteen age- and sex-matched controls who met the same criteria but without glaucoma were recruited through word-of-mouth and flyers.

### Protocol

Participants were consented and evaluated on the same day. This study was carried out in accordance with the recommendations of Institution’s Review Board, Augusta University, Augusta, GA, United States; with written informed consent from all subjects. All subjects gave written informed consent in accordance with the Declaration of Helsinki. Demographic and driving data such as age, education, driving experience, and annual mileage were collected. The Trail Making Test A and B (TMT A & B) (Reitan, 1955; Arbuthnott and Frank, 2000; Sanchez-Cubillo et al., 2009; Kelty-Stephen et al., 2016) and Montreal Cognitive Assessment (MOCA) (Nasreddine et al., 2005) were administered. TMT A is a paper-and-pencil test of information processing and visuomotor tracking in which participants were required to connect 25 circles in an increasing order (1, 2, 3, 4, etc.). TMT B, which additionally tested shifting of attention, required participants to connect 25 circles containing either numbers of letters in alternating order (1, A, 2, B, etc.) (Reitan, 1955; Arbuthnott and Frank, 2000; Sanchez-Cubillo et al., 2009; Kelty-Stephen et al., 2016). The time to complete each test and the number of errors was recorded. MOCA is a comprehensive assessment of cognitive functions that was scored on an ordinal scale ranging from 0 to 30 (Nasreddine et al., 2005).

### Vision Tests

The vision screening apparatus from Keystone view (Visionary Software version 2.0.14) was used as a general screen for binocular visual acuity (20/x) and horizontal field of view (in degrees). The Humphrey Visual Field Analyzer (Humphrey Instrument, Dublin, CA, United States) SITA Fast 24-2 was used for monocular visual field testing and had been established previously with standardized protocols and test–retest reliability (Advanced Glaucoma Intervention Study Investigators, 1994). Mean deviation (MD) corrected absolute values and pattern standard deviation (PSD) were used as outcome measures.

### Driving Simulator and Conditions

**FIGURE 1:**
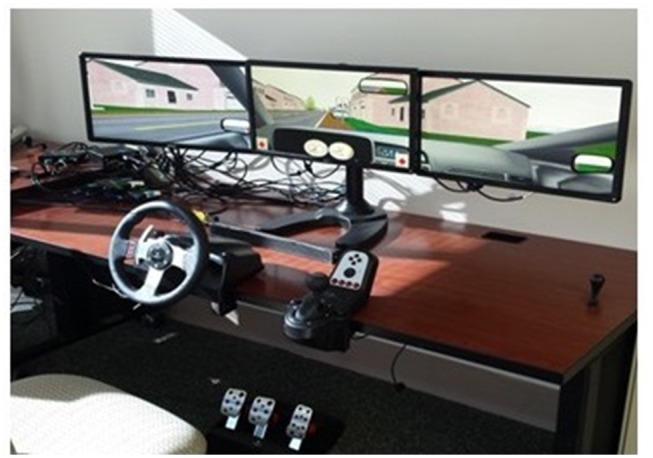
Low-fidelity driving simulator (STISIM drive).

A low fidelity (desktop model) driving simulator with images generated using the STISIM Drive software (STI, Inc., Hawthorne, CA, United States) and displayed on three 22-inch DELL^®^ computer screens was used to measure binocular functional visual field (**Figure 1**). The three screens provided a horizontal field of view of 100° and a vertical field of view of 20°. Participants drove through all simulated scenarios using a Logitech^®^ steering wheel and pedals that were connected to the simulator system. Each condition followed the same protocol. After standardized auditory and written instructions, participants completed a practice trial of 45 s, followed by the actual evaluation of 13 min. The ambient luminance in the darkened room was on average 0.40 cd/m^2^. The display luminance of condition 1 was on average 21.64 cd/m^2^, and 24.56 cd/m^2^ for conditions 2 and 3.

**FIGURE 2:**
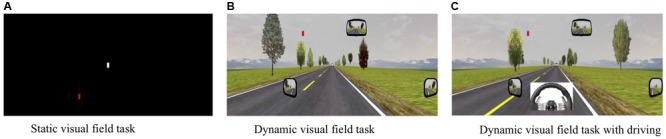
**(A–C)** Three different visual field conditions used in the study.

**FIGURE 3:**
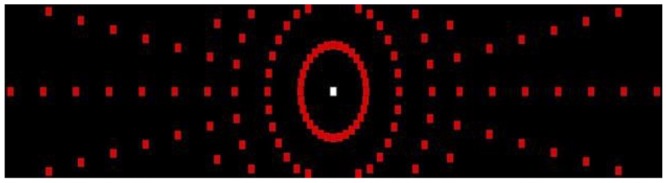
Visual field targets expanding 100° of horizontal field of view and 20° of vertical field of view.

*Simulator Test Condition 1* (**Figure 2A**) involved static visual field testing on a black background. Participants were to focus on a central fixation point (white square, RGB 255/255/255) at eye height in the middle of a black screen (RGB 0/0/0). Participants were instructed to press a button on the steering wheel with their right thumb as soon as they localized a red square (RGB 255/0/0) that appeared in the periphery of the black screen. The size and dimensions of the squares (2.0 cm × 2.0 cm) were carefully determined after review of the literature (Bentley et al., 2012). The red square appeared at different degrees of eccentricity (5–100° of horizontal angle and 5–20° of vertical angle, each in 5° increments) on 8 line coordinates at various degrees of radial angles (0–337.5° in 22.5° increments). Horizontal and vertical angles were defined as the angle between the line perpendicular to the screen through the origin of gaze and the line through the center of the symbol and the origin of gaze. Overall, 114 symbols were presented at random time intervals (between 0.5 and 2 s) and at an unpredictable amplitude (**Figure 3**).

*Simulator Test Condition 2* (**Figure 2B**) involved a dynamic visual field task with no active driving to evaluate the effect of optic flow on functional visual field. Like condition 1, participants were requested to press the thumb button as soon as they localized the peripheral target. However, the background was changed to a dynamic driving scene with an automatic pilot of 45 mph. A white lead vehicle with the same dimensions as the central white square in condition 1 was used as fixation point.

*Simulator Test Condition 3* (**Figure 2C**) involved the same dynamic visual field task but with the participant actively driving to evaluate the additional impact of psychomotor activity on functional visual field. In this condition, the participant was requested to drive the car on a straight road at a constant speed of 45 mph. The participant focused on a lead car and there were auditory speed warnings if the driver drove at 5 mph above or below the stipulated speed. In addition to focusing on the lead car, the participant pressed the thumb button whenever a red square appeared on the screen.

Computer-generated measures of number of correct responses (accuracy) and the response time to the peripheral target in all three conditions were the primary outcome measures. Automatically generated driving data from condition 3 such as time spent and distance driven over the speed limit, time spent and distance driven over the center lane, mean lateral position and speed, and standard deviation of lateral position and speed were used as secondary outcome measures at 60 Hz.

### Useful Field of View Test (UFOV^®^)

The UFOV^®^ is a binocular functional visual field test involving three subtests that increased in cognitive demand with each subsequent subtest. In the speed of processing subtest, the participant had to identify a target presented in the central vision. In the divided attention subtest, the participant had to identify the target presented in the central vision along with a concurrent peripheral target localization task. In the selective attention subtest, the participant performed similar tasks as in divided attention subtest. However, the target displayed in the periphery was embedded in distracters. All subtests were measured in milliseconds. More detailed description of the UFOV^®^ test has been reported elsewhere (Ball et al., 1993; Tatham et al., 2015).

### Fixation Stability and Cognitive Workload

The FOVIO eye tracker (Seeing Machines, Inc., Canberra, ACT, Australia) was used to confirm fixation of the central point when peripheral targets were presented to ensure reliability of visual field testing. The percentage gaze time on the central fixation target across three conditions was used as an outcome variable. The cognitive workload, i.e., the amount of mental effort indexed through TEPR, was also recorded at 60 Hz by analyzing the changes in raw pupil size of the left eye, while adjusting for individual differences in pupil size, lighting and accommodation (Marshall, 2000). Although TEPR is sensitive to cognitive workload and task difficulty in working memory, it is not accurate to detect complexity of sentences in older adults (Piquado et al., 2010). Calibration of pupils takes about 2 min. TEPR has been found to correlate well with other indices of neural activity, such as electro-encephalogram and functional magnetic resonance imaging (Ranchet et al., 2017b). The TEPR scores were transformed into a continuous scale of cognitive workload. This Index of Cognitive Activity (ICA) ranges from 0 to 1, with greater values indicating more cognitive workload. The resulting ICA scores are thought not to be subject to practice effects, education, race, and sex (Marshall, 2007).

### Data Analysis

Data were checked for normal distribution using the Kolmogorov–Smirnov statistic. Results from the normality testing enabled us to use non-parametric analyses for all hypotheses. Friedman analysis and *post hoc* pairwise comparisons using the Wilcoxon Signed Rank Test were conducted for hypothesis 1. The Wilcoxon Rank Sum test was conducted for hypothesis 2 and 3 and to examine between group differences in demographics, UFOV^®^ metrics and driving simulator measures. Since the ICA data was normally distributed, general linear models were used to verify the main effects of group (glaucoma vs. healthy controls) and condition (1, 2, and 3), and the interaction effect of group by condition, on cognitive workload. *Post hoc* pairwise comparison was employed to investigate differences in main and interaction effects. Chi-square analysis was performed to determine the effect of cognitive demand on the eccentricity of missed responses across the three conditions for both groups. All analyses were conducted with SPSS version 23. *p*-values of less than 0.05 were considered significant.

## Results

### Comparison of Demographic, Clinical, and Visual Characteristics

Sixteen participants had bilateral open-angle glaucoma and four had unilateral open-angle glaucoma. The differences between the glaucoma and healthy control groups in demographics, clinical and visual field measures are presented in **Table 1**. Both groups differed significantly in TMT B, mean deviation and pattern standard deviation of left eye and mean deviation of right eye derived from HVF.

**Table 1:** Demographics, clinical and visual measures between the glaucoma (*n* = 20) and healthy control (*n* = 13) groups.

Variable	Glaucoma		Controls		W value^∗^	*p*-value
	Median	Q1–Q3	Median	Q1–Q3		
**Demographics**				
Age (years)	62.50	59–71	57	53–70	178	0.11
Education (years)	13.50	12–16	12	12–14	197.50	0.39
Driving experience (years)	46.50	40.50–51.50	42	37–54	205.50	0.58
Annual mileage (miles/year)	12000	5500–15500	12500	8000–18200	232	0.70
**Clinical measures**				
TMT A (seconds)	36	28.50–48.50	29.50	26.50–40.57	156.50	0.11
TMT B (seconds)	124.50	81.50–190.50	72	57.50–96.00	145.50	0.04^∗∗^
MOCA (0–30)	26.50	24.50–29.00	27.50	27.00–28.50	216.50	0.47
**Visual measures**				
Visual acuity (20/x)	30	20–40	25	20–30	137	0.28
Total visual field (0–170°)	170	155–170	170	170–170	183	0.19
Humphrey left MD	5.04	1.45–13.24	0.90	0.53–2.17	252	0.006^∗∗^
Humphrey left PSD	2.84	2.00–9.20	1.51	1.32–2.30	116	0.003^∗∗^
Humphrey right MD	2.90	1.20–10.72	0.61	0.08–2.51	258	0.02^∗∗^
Humphrey right PSD	1.93	1.60–8.63	1.90	1.51–2.34	173.50	0.35

*^∗^ indicate Wilcoxon Rank sum test; ^∗∗^ indicate *p*-value < 0.05; MOCA, Montreal Cognitive Assessment; TMT A and TMT B, trail making test A and B; MD, mean deviation; PSD, pattern standard deviation.*

### Effect of Cognitive Demand on Functional Visual Field

Within group comparisons showed a significant effect of cognitive demand on functional visual field performance in both groups (**Table 2**). *Post hoc* comparisons showed that both groups responded less accurately and slower in conditions 2 and 3 compared to condition 1 (*p* < 0.05). Between groups comparisons did not reveal significant differences in the performance on the static visual task (condition 1, **Table 2** and Supplementary Figures 1A,B). However, the glaucoma group responded less accurately and slower in the dynamic visual field task without active driving (condition 2) and with active driving (condition 3) compared with healthy controls (*p* < 0.05) (**Table 2** and Supplementary Figures 1A,B). Pairwise comparisons showed that adding visual flow to the visual field test without (C2–C1) or with active driving (C3–C1) affected accuracy on the functional visual field worse in the glaucoma group than in healthy controls (**Table 2**). No such effects were observed on response time.

**Table 2:** Functional visual field performance of the glaucoma (*n* = 20) and healthy control (*n* = 13) groups.

	Condition 1 (C1)	Condition 2 (C2)	Condition 3 (C3)	Within group *p*-value^‡^	Pairwise comparisons^$^		
**Correct responses**							
Glaucomaˆ	114 (113–114)	111 (106–112)	111.50 (104–112)	0.001^∗^	C2–C1^∗^	C3–C1^∗^	C3–C2
Controlsˆ	114 (114–114)	112 (111–114)	113 (112–114)	0.02^∗^	C2–C1^∗^	C3–C1^∗^	C3–C2
Between group *p*-value^§^	0.20	0.01^∗^	0.01^∗^		0.05	0.02^∗^	0.047^∗^
**Response time (s)**							
Glaucomaˆ	0.52 (0.49–0.60)	0.63 (0.56–0.82)	0.76 (0.64–0.95)	0.001^∗^	C2–C1^∗^	C3–C1^∗^	C3–C2^∗^
Controlsˆ	0.47 (0.43–0.52)	0.58 (0.52–0.60)	0.64 (0.57–0.73)	0.001^∗^	C2–C1^∗^	C3–C1^∗^	C3–C2^∗^
Between group *p*-value^x^	0.08	0.01^∗^	0.04^∗^		0.17	0.34	0.73

*ˆindicate values in median (Q1–Q3); ^∗^indicate *p*-value < 0.05; Response time in seconds; ^§^indicate *p*-value from Wilcoxon rank sum test; ^‡^indicate *p*-value from Friedman Test; ^$^Wilcoxon signed rank test.*

No differences in percentage gaze time on the central fixation were found between both groups, indicating that both groups spent an equal amount of time looking at the central target (*p* > 0.05, data not shown). Finally, with increased cognitive demand, drivers with glaucoma missed more responses at greater angles of eccentricity (χ^2^ = 32.11, *p* < 0.05, Supplementary Table 1). No such effect was seen in healthy controls.

### Cognitive Workload

**FIGURE 4:**
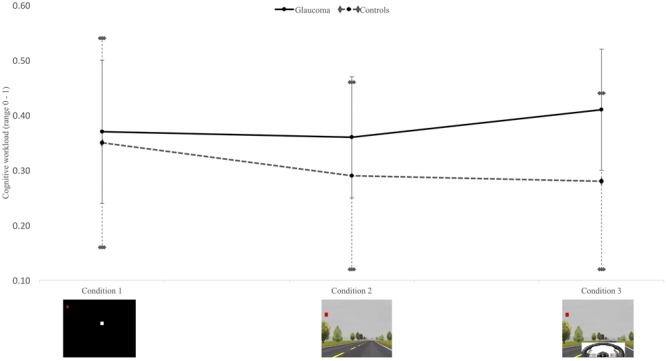
Mean and standard deviations for cognitive workload between the glaucoma (*n* = 20) and healthy control (*n* = 13) groups across three conditions; between group effect *p* = 0.02.

General linear models further revealed a significant effect of glaucoma (*F* = 5.45; *p* = 0.02) on the cognitive workload, as indexed by increased ICA (**Figure 4**). Overall, individuals with glaucoma exhibited greater cognitive workload across all three conditions compared to controls [condition 1: ICA glaucoma mean (SD), 0.37 (0.13); controls, 0.35 (0.19); condition 2: ICA glaucoma, 0.36 (0.11); ICA controls, 0.29 (0.17); condition 3: ICA glaucoma, 0.41 (0.11); ICA controls 0.28 (0.16)]. Within group analyses revealed no significant differences in the cognitive workload (*F* = 0.38; *p* = 0.68). Likewise, the interaction effect of group by condition was not significant (*F* = 1.03; *p* = 0.36).

### UFOV Tasks

A similar pattern of effect of cognitive demand was observed for the UFOV^®^. Within group analyses revealed that both groups showed slower speed of processing as the tasks became more difficult (*p* < 0.05, Supplementary Table 2). Between group differences in UFOV^®^ metrics demonstrated that the glaucoma group was significantly slower while performing the divided and selective attention tasks than the healthy controls (*p* < 0.05, Supplementary Table 2). The addition of a car symbol in a non-cluttered (divided attention) and cluttered (selective attention) periphery disproportionally worsened speed of processing in the glaucoma group compared to the control group (*p* < 0.05, Supplementary Table 2).

### Comparison of Driving Simulator Characteristics

Analyzing the driving simulator performance (condition 3) of both groups revealed that the glaucoma group was significantly different from the control group in time spent (*p* = 0.05) and distance (*p* < 0.05) driven over the lane (**Table 3**). However, no significant differences were observed between these groups in other driving simulator measures.

**Table 3:** Driving simulator performance of the glaucoma (*n* = 20) and healthy control (*n* = 13) groups during condition 3.

	Glaucoma		Controls		W value^∗^	*p*-value
	Median	Q1–Q3	Median	Q1–Q3		
Time spent over the speed limit (seconds)	48.4	31.0–59.4	52.5	35.3–61.0	285	0.68
Distance driven over the speed limit (feet)	51.3	61.0–33.7	54	36.9–62.6	287	0.75
Time spent over the center lane (seconds)	4.2	3.1–6.5	1.1	0.6–3.5	125	0.05^∗∗^
Distance driven over the center lane (feet)	4.6	3.2–7.4	1.1	0.6–3.5	114	0.01^∗∗^
Mean lateral position (feet)	5.3	4.6–5.8	5.9	5.0–6.2	266	0.22
Mean speed (miles/hour)	44.9	44.4–45.1	44.9	44.5–45.1	281	0.56
Standard deviation lateral position (feet)	1.2	0.8–1.3	0.9	0.8–1.4	163	0.75
Standard deviation speed (miles/hour)	1.3	1.1–1.9	1.3	1.1–1.4	155	0.50

*^∗^ indicate Wilcoxon Rank sum test; ^∗∗^ indicate *p*-value ≤ 0.05.*

## Discussion

This study is one of the very few studies to investigate the effects of cognitive demand on the functional visual field of drivers with glaucoma in comparison to healthy controls. Our findings support our hypotheses that an increase in cognitive demand reduced the functional visual field performance both in drivers with glaucoma as well as healthy controls. However, drivers with glaucoma performed worse on the visual field task, especially when dynamic visual flow was added. Furthermore, the study findings demonstrated that an increase in cognitive demand disproportionately worsened the functional visual field performance of drivers with glaucoma compared with healthy controls. Our results therefore suggest that visual field testing for activities that require timely detection of stimuli in a highly dynamic and rapidly changing environment such as driving should consider the participants’ physiological visual field, their cognitive capacity, and the cognitive demand of the task to fully appreciate the impact of any visual field loss performance.

Drivers with glaucoma differed significantly from healthy controls in the functional visual field while reacting to an increase in cognitive demand in a driving simulator. Although the addition of visual flow affected the functional field of view similarly in both groups, participants with glaucoma performed disproportionally worse when the psychomotor component of operating the steering wheels and pedals was added. The allocation of cognitive resources to focusing on the central target, concentrating on identifying the peripheral target in a cluttered environment, while maintaining control over the vehicle, resulted in disproportionately greater cognitive workload in drivers with glaucoma. As a result, drivers with glaucoma identified fewer symbols than controls in the functional visual field tests compared to their baseline performance on the static visual field test. In particular, the symbols in the periphery became more difficult to detect with increased cognitive demand. Our findings support the results of Prado Vega et al. (2013) that also showed drivers with glaucoma to detect fewer peripheral stimuli.

The relationship between increased cognitive demand of the task and reduction in functional visual field has been studied previously using the UFOV^®^ test (Gracitelli et al., 2015; Tatham et al., 2015; Lee et al., 2017). In those studies, the divided attention subtest of the UFOV^®^ showed to correlate best with motor vehicle crashes (MVC) in drivers with glaucoma (Gracitelli et al., 2015; Tatham et al., 2015; Lee et al., 2017). Tatham et al. (2015) observed that individuals with glaucoma who had a history of MVC reported reduced divided attention metrics of the UFOV^®^ test than those drivers with no MVC history, suggesting that increased cognitive demand shrunk the functional visual field, and in turn, impacted driving safety. Our study confirms that drivers with glaucoma perform worse on the UFOV^®^, but only in the dual task conditions of divided attention and selective attention.

The increase in cognitive demand did not only affect their performance on the visual field test, drivers with glaucoma also exhibited poorer performance in vehicle control. Participants with glaucoma drove longer over the center lane and crossed the center lane for a greater distance than did controls. Previous studies showed that driving performance of participants with glaucoma was significantly reduced with higher number of collisions than the age-matched controls on a driving simulator (Kunimatsu-Sanuki et al., 2015). This was in spite of the fact that tasks used in that particular study were neither cognitively demanding nor did they evaluate visual field tasks in a functional setting. Participants were asked to follow simple traffic signals or stop signs, obstacle avoidance in terms of vehicles or children rushing out from the sides (Kunimatsu-Sanuki et al., 2015). By contrast, Prado Vega et al. (2013) did not find significant differences between groups in the performance of primary tasks such as lane keeping and obstacle avoidance. Yet, drivers with glaucoma in their study exhibited increased steering activity, suggesting more difficulty performing the driving task (Prado Vega et al., 2013).

In addition to decrements in performance on the visual field tasks and the driving tasks, participants with glaucoma also showed increased cognitive workload across all three conditions. This finding suggests that drivers with glaucoma had to concentrate harder to detect the visual field symbols in all three conditions. Our ICA was based on TEPR, a real-time physiological measure of mental effort. To our knowledge, this is the first time that a neurophysiological measure of cognitive workload was used to determine mental effort during functional visual field tasks in glaucoma. Prado Vega et al. (2013) found no significant differences in self-reported cognitive workload between drivers with glaucoma and controls in four simulator conditions. However, self-report ratings depend on the perception of the individuals, whereas the ICA is thought to be a reliable, objective estimate of cognitive workload (Marshall, 2007). Whereas Prado Vega et al. (2013) found a significant effect of cognitive demand on subjective cognitive workload, our study did not show a linear relationship between cognitive demand and cognitive workload. Psychophysiological studies have demonstrated that cognitive workload increases as a function of cognitive demand, until a tipping point is reached where the task becomes too difficult to complete (Beatty, 1982). The decrements in accuracy and response time with increased cognitive demand may have resulted in cognitive overload. As a result, no within group effects on cognitive workload were observed in our study. Further research is warranted to confirm the usefulness of pupillometry as an objective, real-time measure of cognitive workload in glaucoma.

The results of the study should be considered preliminary. We found that the functional visual field of the participant with glaucoma with slight to moderate visual impairment was altered when driving in a highly-cluttered environment such as driving. However, care should be taken when generalizing these findings to a larger patient population since our sample size was small. Future studies should include a larger sample of drivers with glaucoma with various severity of visual impairment to confirm our findings. Such studies should also aim at generalizing the driving simulator findings to real-life on-road driving ability because our dynamic driving simulator task only included a car following task.

## Conclusion

Cognitive demand, especially in a functional context such as driving, significantly reduced the functional field of view of individuals with glaucoma. The disproportionate impact of cognitive demand on functional visual field in glaucoma was more evident when cluttered visual flow was added than when a psychomotor activity was added to the visual field task. Our findings suggest that visual field testing to determine eligibility for driving resumption in glaucoma needs to be conducted in a cluttered, driving-related setting to fully appreciate the cognitive demands of real-world driving.

## Author Contributions

VG: data analyses and drafted the manuscript; AA: valuable suggestions and manuscript revision; MR: suggestions and manuscript revision; KB: suggestions and manuscript revision; HD: suggestions and manuscript revision, drafting the manuscript, help with data analyses and provided valuable suggestions.

## Conflict of Interest Statement

The authors declare that the research was conducted in the absence of any commercial or financial relationships that could be construed as a potential conflict of interest.
