# Supplementary material for: Effect of Cognitive Demand on Functional Visual Field Performance in Senior Drivers with Glaucoma
Source: Front Aging Neurosci. 2017 Aug 30;9:286. doi: 10.3389/fnagi.2017.00286 (PMC5582159; doi:10.3389/fnagi.2017.00286)
Supplement: Supplementary file 1 [file Data_Sheet_1.DOCX]

**Title: Effect of Cognitive Demand on Functional Visual Field Performance in Senior Drivers with Glaucoma**

**Viswa Gangeddula¹, Maud Ranchet², Abiodun Akinwuntan¹ Kathryn Bollinger^3^, Hannes Devos¹˒***

¹ Department of Physical Therapy and Rehabilitation Science, University of Kansas Medical Center, Kansas City, Kansas, USA

² Laboratory Ergonomics and Cognitive Sciences applied to Transport, Lyon, France.

^3^ Department of Ophthalmology, Medical College of Georgia, Augusta University, Augusta, Georgia, USA

*Correspondence:

Hannes Devos, Email: [hdevos@kumc.edu](mailto:hdevos@kumc.edu)

1. Supplementary Figures and Tables

1.1 Supplementary Figures


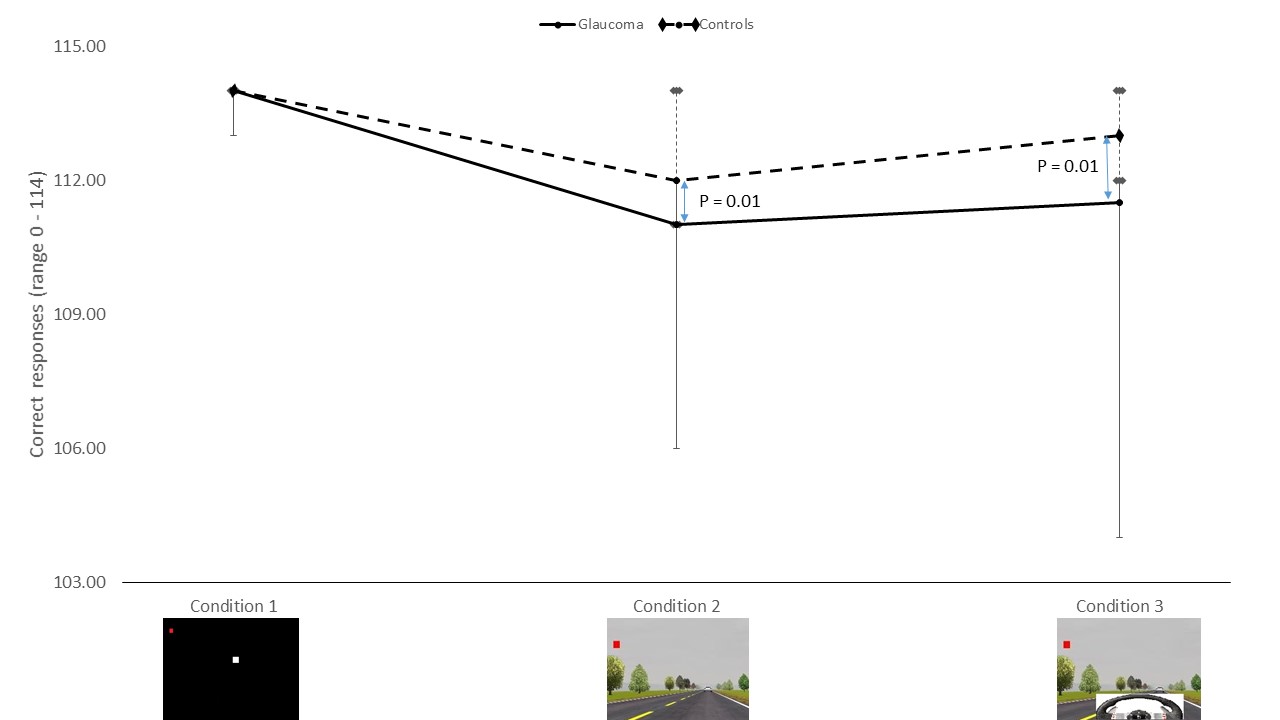


Supplementary figure 1(A): Median, Q1 and Q3 scores of correct responses for the glaucoma (n = 20) and healthy controls (n = 13) across three conditions.


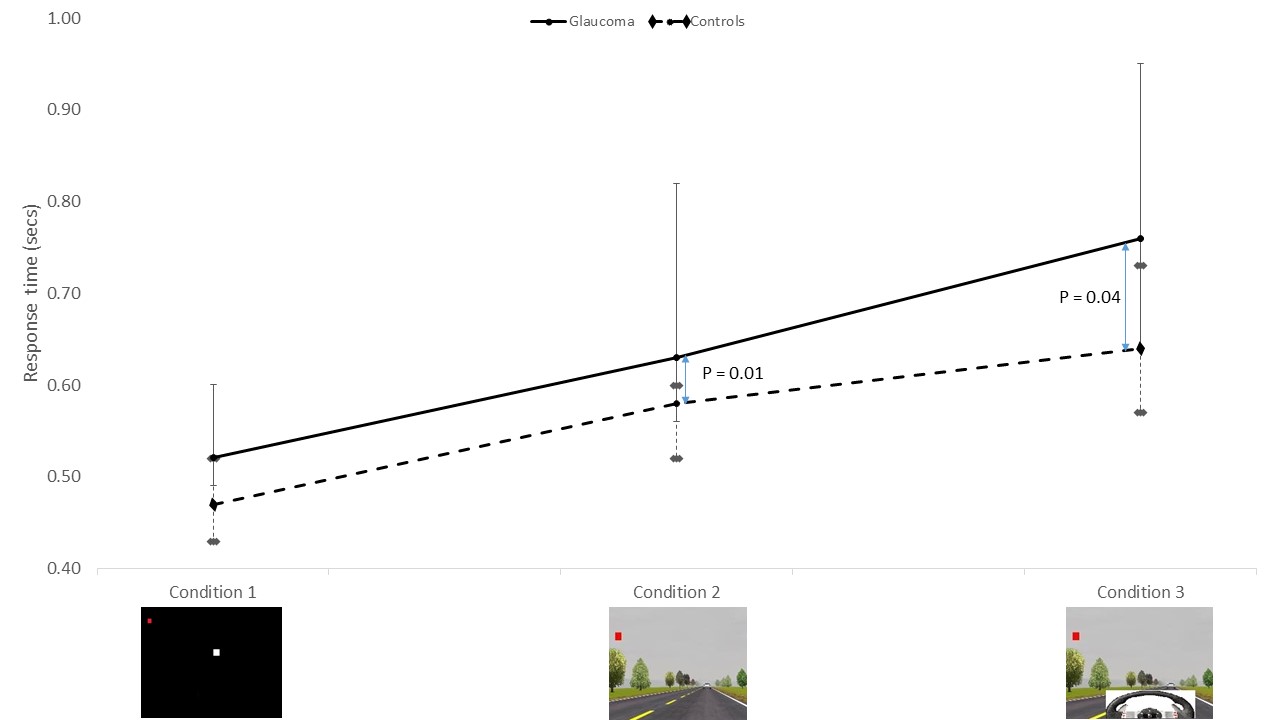


Supplementary figure 1(B): Median, Q1 and Q3 scores of response time for the glaucoma (n = 20) and healthy controls (n = 13) across three conditions.

1.2 Supplementary Tables

Supplementary Table 1. Chi-square analysis of missed responses at various eccentricity for the Glaucoma group (n=20).

| Eccentricity | Condition 1 | Condition 2 | Condition 3 | Grand Total |
| --- | --- | --- | --- | --- |
| 5 | 7 | 3 | 9 | 19 |
| 10 | 12 | 22 | 17 | 51 |
| 15 | 7 | 15 | 13 | 35 |
| 20 | 5 | 7 | 6 | 18 |
| 25 | 3 | 3 | 3 | 9 |
| 30 | 1 | 3 | 3 | 7 |
| 35 | 2 | 5 | 8 | 15 |
| 40 | 2 | 17 | 9 | 28 |
| 45 | 1 | 25 | 29 | 55 |
| 50 | 4 | 22 | 13 | 39 |
| Grand Total | 44 | 122 | 110 | 276 |

χ² = 32.112, p < 0.05

Supplementary Table 2. Useful Field of View (UFOV) tasks of the Glaucoma (n = 20) and healthy control group (n = 13).

|  | PS (ms) | DA (ms) | SA (ms) | Within group p-value ǂ | Pairwise comparisons | | |
| --- | --- | --- | --- | --- | --- | --- | --- |
| **Group** |  |  |  |  |  | | |
| Glaucoma^ | 16.70 (16.70 - 50.00) | 183.20 (30.10 - 440.00) | 500.00 (200.10 - 500.00) | 0.001* | DA - PS | SA - PS | SA - DA |
| Controls^ | 16.70 (16.70 - 16.70) | 69.90 (16.70 - 123.50) | 170.00 (83.50 - 273.30) | 0.001* | DA - PS | SA - PS | SA - DA |
| Between group p-value § | 0.22 | 0.04* | 0.01* |  | 0.04* | 0.01* | 0.90 |

^ indicate values in median (Q1 – Q3); PS, DA and SA indicate processing speed, divided attention, selective attention respectively in milliseconds; § indicate p-value from Wilcoxon rank sum test; ǂ indicate p-value from Friedman Test; pairwise comparisons from Wilcoxon signed rank tes
